# Supplementary material for: Conformational basis of subtype-specific allosteric control of NMDA receptor gating
Source: bioRxiv. 2024 Feb 11:2024.02.10.579740. Preprint. [Version 1] doi: 10.1101/2024.02.10.579740 (PMC10871359; doi:10.1101/2024.02.10.579740)
Supplement: Supplement 1 [file media-1.pdf]

Supplemental Figures

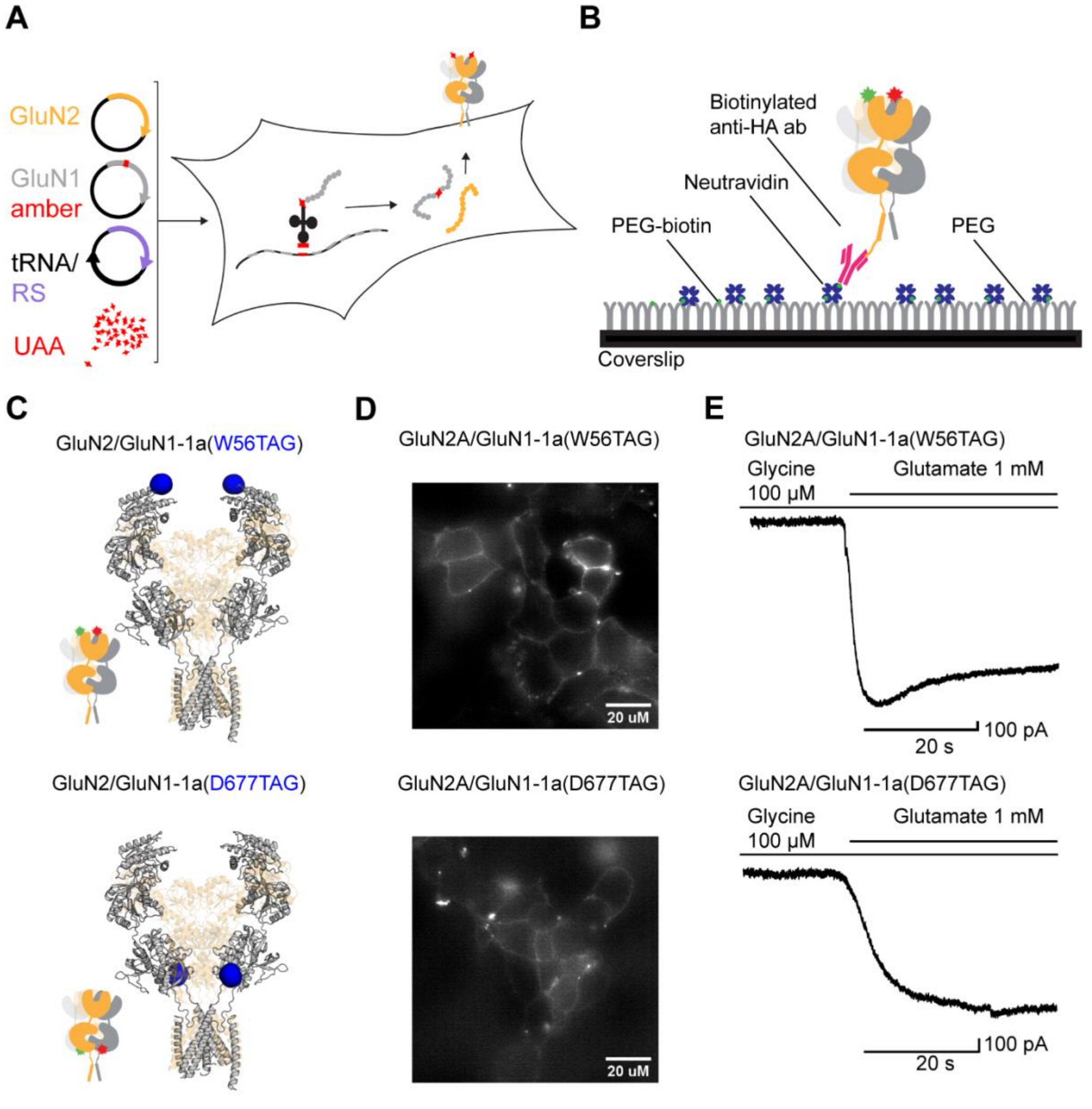

Figure S1. Expression and labeling of TCOK-incorporated NMDA receptors (A) Schematic of expression of NMDA receptors with site-specifically incorporated unnatural amino acids. Diagram inspired by Klippenstein et al. 2017[1] (B) Schematic of labeled immune-purified NMDA receptor in imaging chamber with GluN2 HA tag bound to biotinylated anti-HA antibody, which is bound to neutravidin which is bound to biotin-PEG (C) Structures (PDB 7EOS[2]) and cartoons depicting GluN1 labeling sites (D) Epifluorescence image of acceptor (tetrazine-AF647) labeled HEK293T cells expressing GluN1(W56TAG)/GluN2A (top) or GluN1(D677TAG)/GluN2A (bottom) receptors. (E) Representative whole cell patch clamp current traces in donor and acceptor labeled GluN1(W56TAG)/GluN2A (top) or GluN1(D677TAG)/GluN2A (bottom) receptors in HEK293T cells in response to pipette application of 1 mM glutamate in the presence of 100 μM glycine.

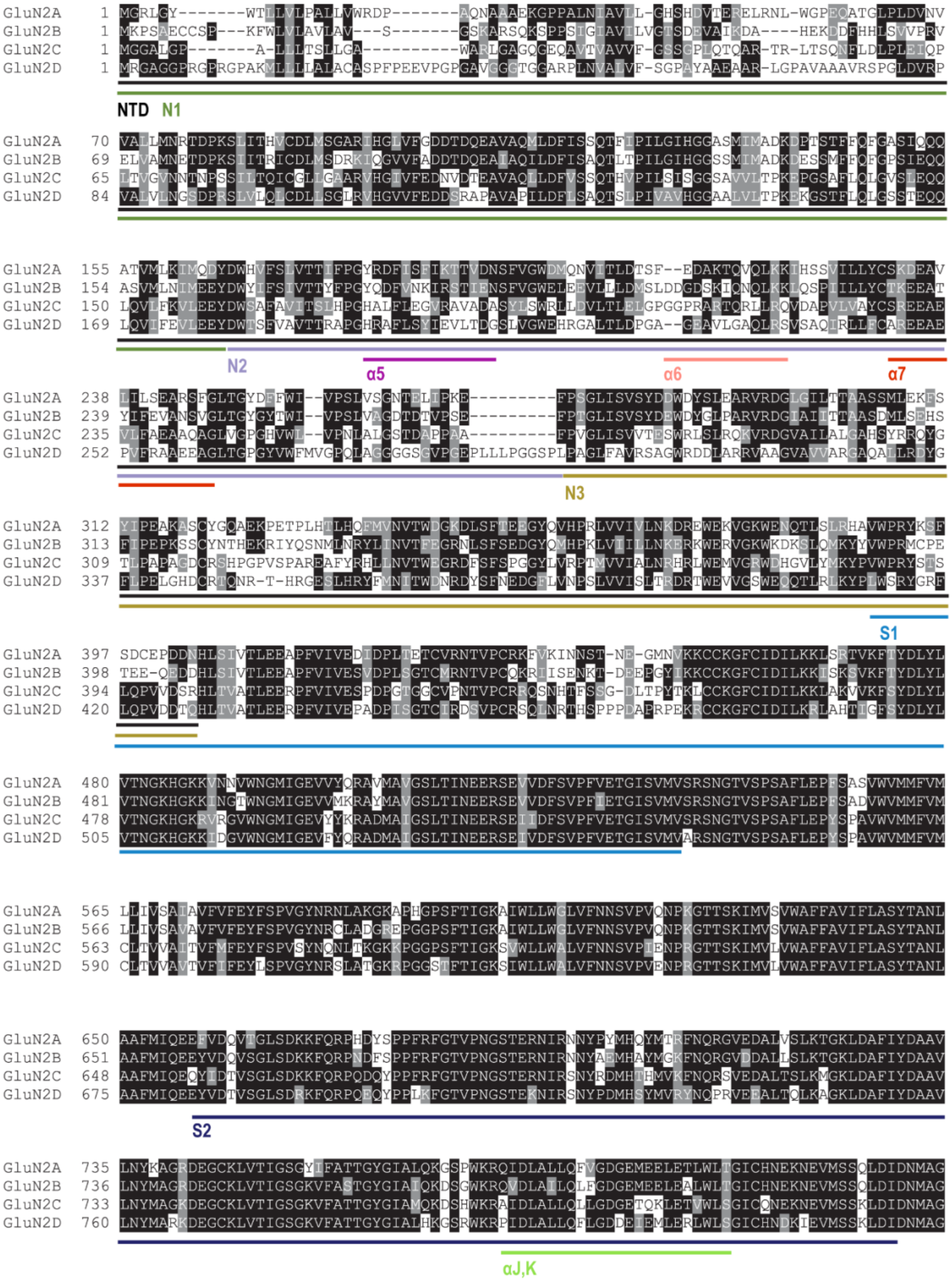

Figure S2. GluN2 sequence alignment Partial GluN2 sequence alignment (T-Coffee[3]) showing the regions that were exchanged in chimeric receptors, shaded with Boxshade.[4]

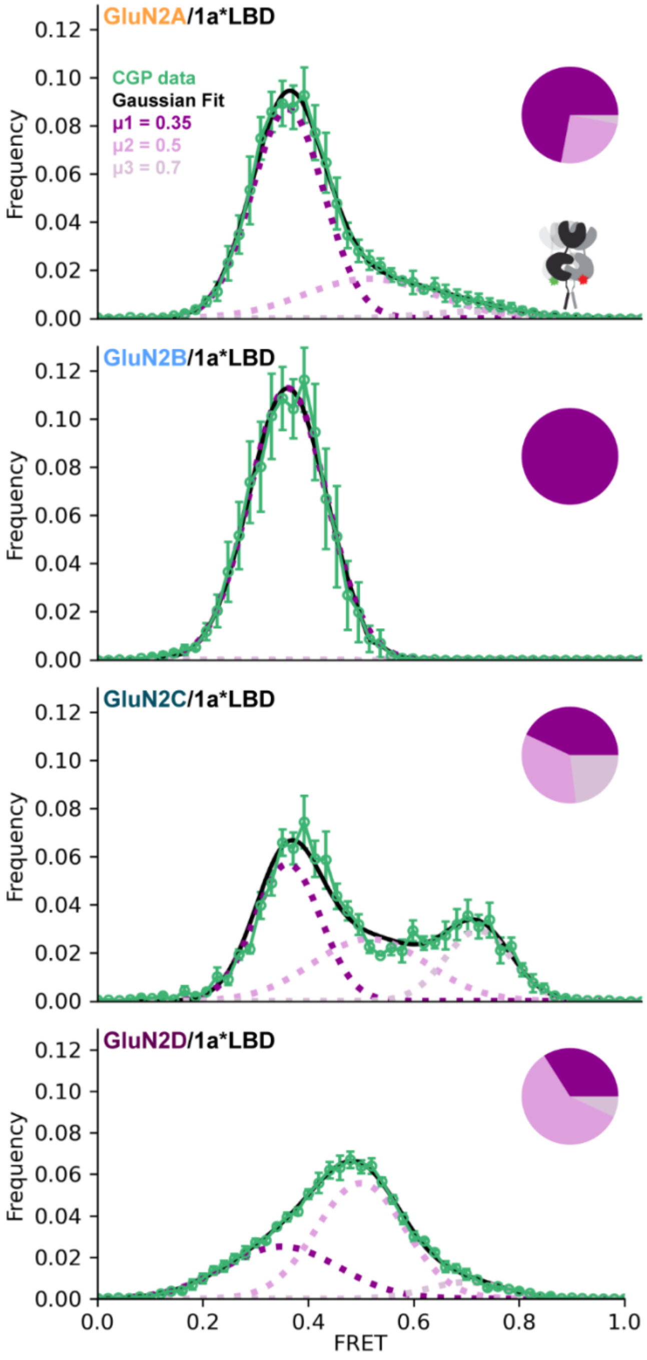

Figure S3. Gaussian fits. Trimodal Gaussian fits (black) of inter-subunit FRET distribution (green, mean +/- S.E.M., from Fig. 1A) between GluN1(D677TAG) LBDs in Apo-like state (zero added glycine, 3 μM GluN1 antagonist CGP78608, zero added glutamate) combined with each of the GluN2 subunits. Individual Gaussians centered at 0.35, 0.5, and 0.7 (dotted, shades of purple) with the corresponding percentages of area of the total fit in pie charts (right insets). Percentages, rounded to the nearest integer for Gaussians centered at FRET = 0.35: 72 (GluN2A), 100 (GluN2B), 43 (GluN2C), 34 (GluN2D); FRET = 0.5: 25 (GluN2A), 0 (GluN2B), 34 (GluN2C), 59 (GluN2D); FRET = 0.7: 3 (GluN2A), 0 (GluN2B), 23 (GluN2C), 7 (GluN2D).

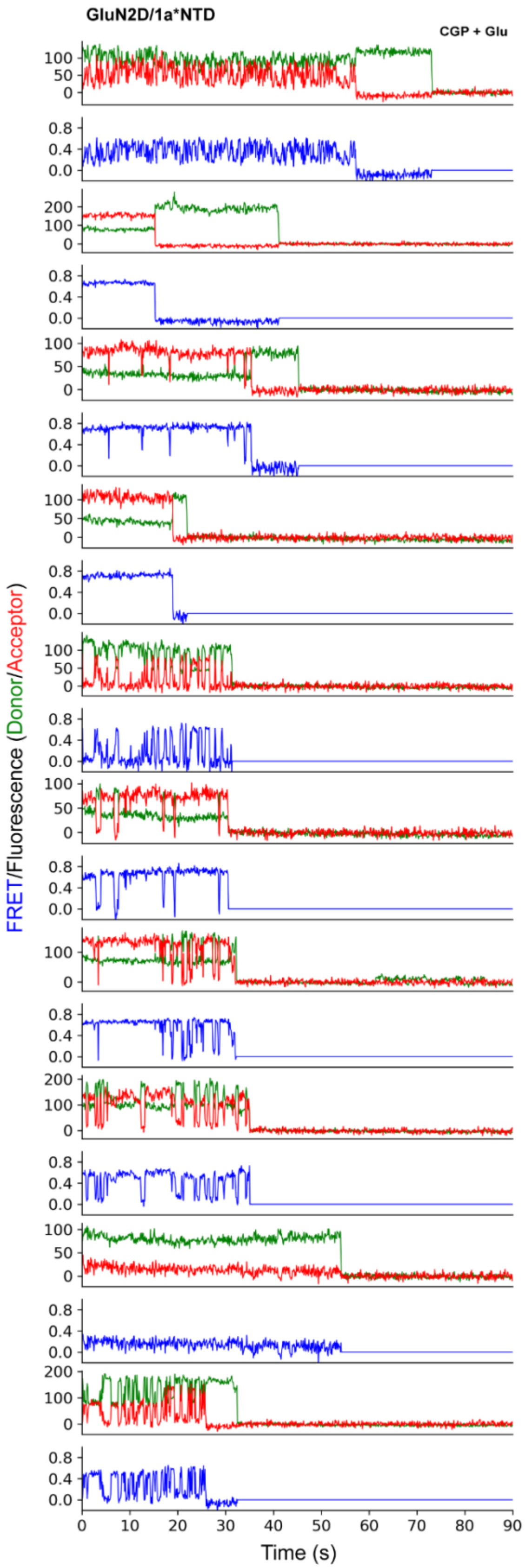

Figure S4. Example traces showing interconversions between splayed, compact and super-compact states Reporting inter-subunit FRET (blue) between GluN1(W56TAG) NTD paired with GluN2D in 3 μM CGP78608 and 1 mM glutamate. These traces are representative of and included in the CGP + Glu histogram in Figure 2H. Donor (Alexa Fluor 555; green) and acceptor (Alexa Fluor 647; red) dyes imaged at 10 fps.

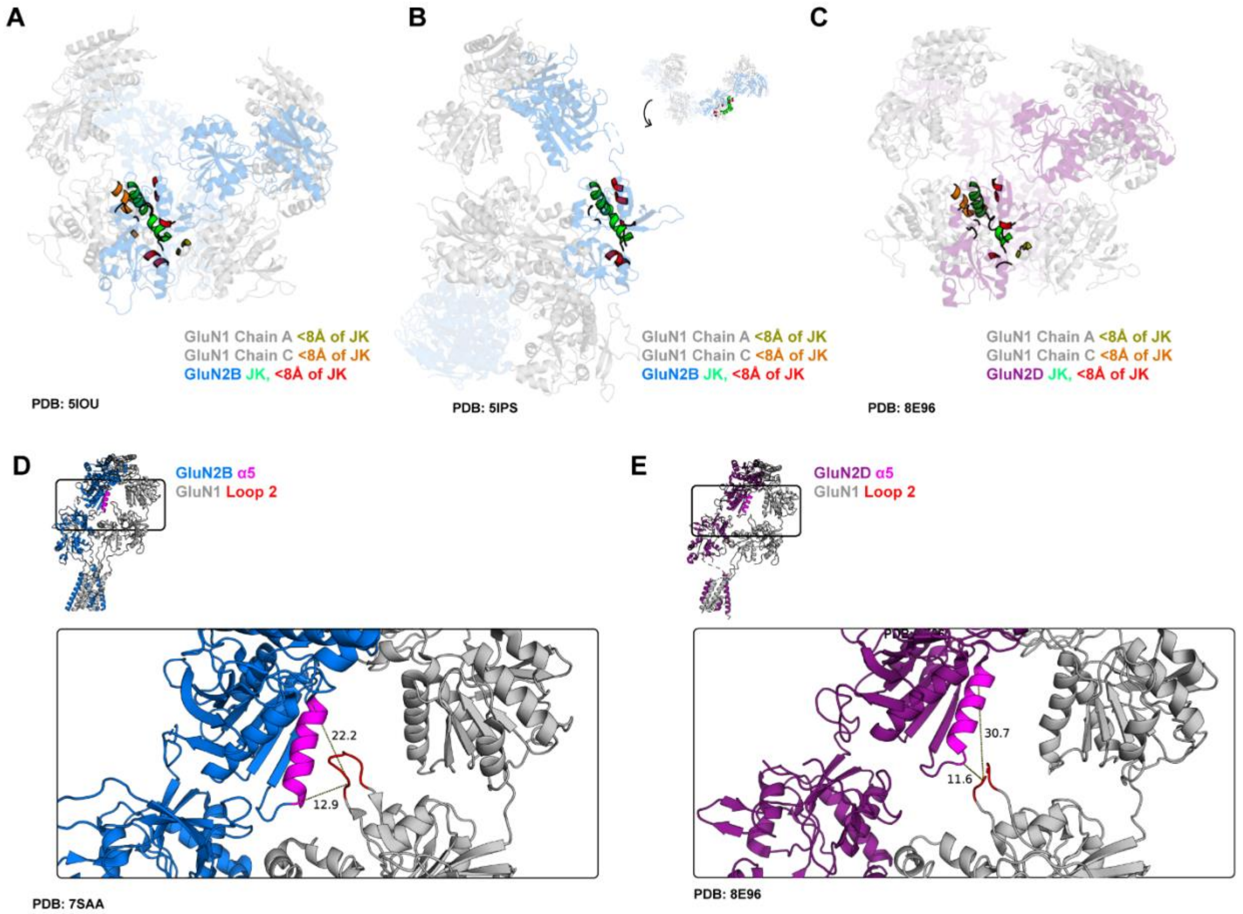

Figure S5. Structures showing interfaces with regions identified in analysis of GluN2B/GluN2D receptor chimeras. (A-C) Highlight of chimeric regions showing exchanged residues in helices J and K (green) and all residues within 8Å of backbone atoms of helices J and K, excluding those immediately adjacent in sequence (segments colored as indicated below structures) in GluN2B splayed (DCKA/D-APV, state 4) structure (PDB: 5IOU[5]) (A), GluN2B compact (glutamate/glycine) structure (PDB: 5IPS[5]) (B) and GluN2D super-compact (glutamate/glycine) structure (PDB: 8E96[7]) (C). (D,E) GluN2 α5 (magenta) – GluN1 loop 2 (red) interface in GluN2B (PDB: 7SAA[6]) (D) and GluN2D (PDB: 8E96[7]) (E). Whole structure above; blown up rectangular region below.

Table S1. Total particle number included in histograms for each condition, by figure.

Figure	Construct	Condition	Total # Particles Included
2	2B/1a(D677TAG)	3uMCGP + 1mM Glu	139
1,2,S3	2B/1a(D677TAG)	3uM CGP	101
1,2	2B/1a(D677TAG)	100uM Gly + 1mM Glu	226
2	2B/1a(D677TAG)	100uM Gly	243
2	2D/1a(D677TAG)	3uMCGP + 1mM Glu	172
1,2,S3	2D/1a(D677TAG)	3uM CGP	220
1,2	2D/1a(D677TAG)	100uM Gly + 1mM Glu	160
2	2D/1a(D677TAG)	100uM Gly	157
2	2A/1a(D677TAG)	3uMCGP + 1mM Glu	138
1,2,S3	2A/1a(D677TAG)	3uM CGP	157
1,2	2A/1a(D677TAG)	100uM Gly + 1mM Glu	116
2	2A/1a(D677TAG)	100uM Gly	246
2	2C/1a(D677TAG)	3uMCGP + 1mM Glu	88
1,2,S3	2C/1a(D677TAG)	3uM CGP	80
1,2	2C/1a(D677TAG)	100uM Gly + 1mM Glu	95
2	2C/1a(D677TAG)	100uM Gly	87
2	2B/1a(W56TAG)	3uMCGP + 1mM Glu	588
1,2	2B/1a(W56TAG)	3uM CGP	577
1,2	2B/1a(W56TAG)	100uM Gly + 1mM Glu	783
2	2B/1a(W56TAG)	100uM Gly	849
2,S4	2D/1a(W56TAG)	3uMCGP + 1mM Glu	266
1,2	2D/1a(W56TAG)	3uM CGP	116
1,2	2D/1a(W56TAG)	100uM Gly + 1mM Glu	290
2	2D/1a(W56TAG)	100uM Gly	229
2	2A/1a(W56TAG)	3uMCGP + 1mM Glu	425
1,2	2A/1a(W56TAG)	3uM CGP	452
1,2	2A/1a(W56TAG)	100uM Gly + 1mM Glu	330
2	2A/1a(W56TAG)	100uM Gly	416
2	2C/1a(W56TAG)	3uMCGP + 1mM Glu	236
1,2	2C/1a(W56TAG)	3uM CGP	276
1,2	2C/1a(W56TAG)	100uM Gly + 1mM Glu	355
2	2C/1a(W56TAG)	100uM Gly	335
3	2B(2D N1)/1a(W56TAG)	3uMCGP + 1mM Glu	206
3	2B(2D N1)/1a(W56TAG)	3uM CGP	215
3	2B(2D N3)/1a(W56TAG)	3uMCGP + 1mM Glu	608
3	2B(2D N3)/1a(W56TAG)	3uM CGP	487
3	2B(2D N1 N3)/1a(W56TAG)	3uMCGP + 1mM Glu	440
3	2B(2D N1 N3)/1a(W56TAG)	3uM CGP	186
3	2B(2D N2 N3)/1a(W56TAG)	3uMCGP + 1mM Glu	312
3	2B(2D N2 N3)/1a(W56TAG)	3uM CGP	373
3	2B/1a(W56TAG)	3uMCGP + 1mM Glu	812
3	2B/1a(W56TAG)	3uM CGP	566
3	2D/1a(W56TAG)	3uMCGP + 1mM Glu	322
3	2D/1a(W56TAG)	3uM CGP	184
3	2B(2D alpha5)/1a(W56TAG)	3uMCGP + 1mM Glu	641
3	2B(2D alpha5)/1a(W56TAG)	3uM CGP	487
3	2B(2D NTD)/1a(W56TAG)	3uMCGP + 1mM Glu	190
3	2B(2D NTD)/1a(W56TAG)	3uM CGP	182
3	2B(2D alpha6)/1a(W56TAG)	3uMCGP + 1mM Glu	671
3	2B(2D alpha6)/1a(W56TAG)	3uM CGP	548

3	2B(2D alpha7)/1a(W56TAG)	3uMCGP + 1mM Glu	592
3	2B(2D alpha7)/1a(W56TAG)	3uM CGP	617
3	2B/1a(W56TAG)	3uMCGP + 1mM Glu	317
3	2B/1a(W56TAG)	3uM CGP	270
3	2D/1a(W56TAG)	3uMCGP + 1mM Glu	169
3	2D/1a(W56TAG)	3uM CGP	145
3	2B(2D S2)/1a(W56TAG)	3uMCGP + 1mM Glu	192
3	2B(2D S2)/1a(W56TAG)	3uM CGP	156
3	2B(2D S1)/1a(W56TAG)	3uMCGP + 1mM Glu	408
3	2B(2D S1)/1a(W56TAG)	3uM CGP	299
3	2B(2D alpha5)/1a(W56TAG)	3uMCGP + 1mM Glu	124
3	2B(2D alpha5)/1a(W56TAG)	3uM CGP	150
3	2B(2D S1 S2)/1a(W56TAG)	3uMCGP + 1mM Glu	207
3	2B(2D S1 S2)/1a(W56TAG)	3uM CGP	270
3	2B(2D alpha 5 S1 S2)/1a(W56TAG)	3uMCGP + 1mM Glu	181
3	2B(2D alpha 5 S1 S2)/1a(W56TAG)	3uM CGP	210
3	2B(2Dalpha5 S1 JK)/1a(W56TAG)	3uMCGP + 1mM Glu	351
3	2B(2Dalpha5 S1 JK)/1a(W56TAG)	3uM CGP	342
3	2B(2Dalpha5 S1)/1a(W56TAG)	3uMCGP + 1mM Glu	314
3	2B(2Dalpha5 S1)/1a(W56TAG)	3uM CGP	244
3	2B(2Dalpha5 S2)/1a(W56TAG)	3uMCGP + 1mM Glu	108
3	2B(2Dalpha5 S2)/1a(W56TAG)	3uM CGP	107
4	2B/1a(W56TAG,489-496GG)	3uMCGP + 1mM Glu	684
4	2B/1a(W56TAG,489-496GG)	3uM CGP	563
4	2B/1a(W56TAG)	3uMCGP + 1mM Glu	348
4	2B/1a(W56TAG)	3uM CGP	380
4	2D/1a(W56TAG)	3uMCGP + 1mM Glu	119
4	2D/1a(W56TAG)	3uM CGP	151
4	2D/1a(W56TAG,489-496GG)	3uMCGP + 1mM Glu	107
4	2D/1a(W56TAG,489-496GG)	3uM CGP	97

References

1. Klippenstein, V., Hoppmann, C., Ye, S., Wang, L., and Paoletti, P. (2017). Optocontrol of glutamate receptor activity by single side-chain photoisomerization. eLife *6*, e25808. 10.7554/eLife.25808.

2. Wang, H., Lv, S., Stroebel, D., Zhang, J., Pan, Y., Huang, X., Zhang, X., Paoletti, P., and Zhu, S. (2021). Gating mechanism and a modulatory niche of human GluN1-GluN2A NMDA receptors. Neuron *109*, 2443-2456.e5. 10.1016/j.neuron.2021.05.031.

3. Notredame, C., Higgins, D.G., and Heringa, J. (2000). T-Coffee: A novel method for fast and accurate multiple sequence alignment. J. Mol. Biol. *302*, 205–217. 10.1006/jmbi.2000.4042.

4. Albà, M. (2000). Making alignments prettier. Genome Biol. *1*, reports2047. 10.1186/gb-2000-1-2-reports2047.

5. Zhu, S., Stein, R.A., Yoshioka, C., Lee, C.-H., Goehring, A., Mchaourab, H.S., and Gouaux, E. (2016). Mechanism of NMDA Receptor Inhibition and Activation. Cell *165*, 704–714. 10.1016/j.cell.2016.03.028.

6. Chou, T.-H., Epstein, M., Michalski, K., Fine, E., Biggin, P.C., and Furukawa, H. (2022). Structural insights into binding of therapeutic channel blockers in NMDA receptors. Nat. Struct. Mol. Biol. *29*, 507–518. 10.1038/s41594-022-00772-0.

7. Chou, T.-H., Kang, H., Simorowski, N., Traynelis, S.F., and Furukawa, H. (2022). Structural insights into assembly and function of GluN1-2C, GluN1-2A-2C, and GluN1-2D NMDARs. Mol. Cell *82*, 4548-4563.e4. 10.1016/j.molcel.2022.10.008.
